# Congenital Rhabdomyosarcoma Presenting as a Neck Mass at Birth

**DOI:** 10.1155/2018/1243436

**Published:** 2018-07-30

**Authors:** Leah E. Waldman, Alex K. Williamson, John B. Amodio, Lee Collins

**Affiliations:** ^1^Department of Radiology, Lenox Hill Hospital of Northwell Health, 100 E. 77th St., New York, NY 10075, USA; ^2^Department of Pathology, Cohen Children's Medical Center of Northwell Heath, 26901 76th Ave., New Hyde Park, NY 11040, USA; ^3^Department of Pediatric Radiology, Long Island Jewish Medical Center of Northwell Heath, 27005 76th Ave., New Hyde Park, NY 11040, USA

## Abstract

Rhabdomyosarcoma is a malignant tumor of the soft tissues which preferentially affects the pediatric population. Neonatal rhabdomyosarcoma is rare, and much of the published literature concerning this entity consists of isolated case reports and small case series. Recent work involving the classification of rhabdomyosarcoma has helped to delineate prognostic information based on gene rearrangements. Here, we present a case of congenital rhabdomyosarcoma seen in utero which manifested as a neck mass at birth and was found to harbor a favorable gene fusion.

## 1. Introduction

Sarcomas are malignant tumors of mesenchymal origin that are much more common in children than adults. Rhabdomyosarcoma is the skeletal muscle variant and is the most common subtype in children, accounting for approximately 3.5% of cancers in children ages 1 to 14 years [1]. The most frequent location is the head and neck, about 35–40% of cases [2]. Other possible locations include the genitourinary tract, limbs, thorax, and retroperitoneum. The majority of cases are sporadic; however, some may be associated with genetic syndromes [3, 4].

According to the WHO classification of soft tissue tumors, there are four distinct subentities of rhabdomyosarcoma and they include embryonal, alveolar, pleomorphic, and spindle cell/sclerosing subtype. The majority of cases fall under the embryonal subtype which comprises botryoid and anaplastic variants. The embryonal subtype has the most favorable prognosis. The next most common subtype is alveolar, representing approximately twenty percent of cases. It tends to have a poorer prognosis because of its metastatic potential and is often diagnosed in the later stages. The pleomorphic subtype occurs almost exclusively in adults [5].

Both CT and MRI can be helpful in diagnosing the tumor and evaluating the extent of disease. Tissue biopsy is required for definitive diagnosis and histologic classification. Treatment is individualized on a per patient basis and usually includes chemotherapy, surgery, and radiotherapy [6].

## 2. Case Report

**Figure 1:**
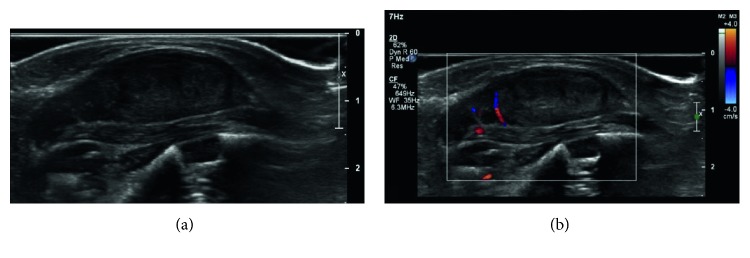
Grayscale (a) and color Doppler (b) sonographic imaging demonstrates a fusiform, heterogeneously hypoechoic mass in the posterior left neck, adjacent to the paraspinal muscle, without significant increased vascularity.

A full-term female infant born to a healthy mother was found at delivery to have a soft tissue mass at the base of the left neck. Of note, the mass was diagnosed as a cystic hygroma in utero. An ultrasound at six days of life demonstrated a solid, ovoid, heterogeneously hypoechoic mass in the posterior left neck, adjacent to the paraspinal muscle measuring 3.2 × 1.4 × 2.1 cm (Figure 1). Because of the location and its ubiquitous nature, the first diagnostic consideration was fibromatosis colli. The initial ultrasound examination aided in localizing the mass external to the scalene muscle, leading us away from this diagnosis. Also included in our differential diagnosis was infantile myofibroma.

**Figure 2:**
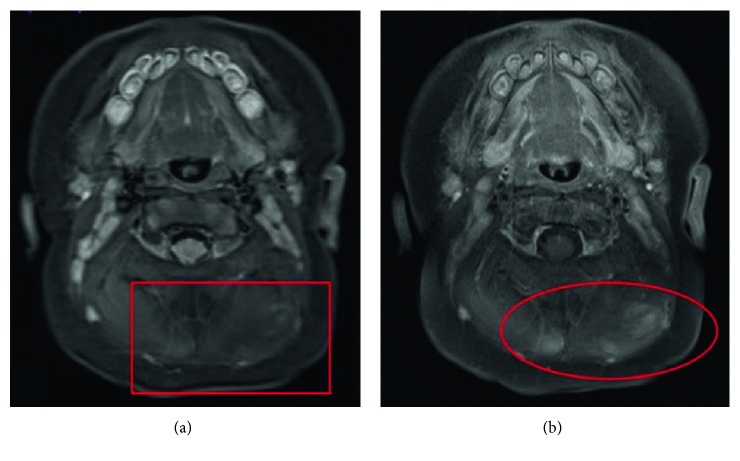
(a) Axial STIR image shows areas of increased T2 signal in the rounded masses within the right and left paraspinal muscles (rectangle). (b) Axial T1 postcontrast image shows mild enhancement within the rounded masses (circle).

**Figure 3:**
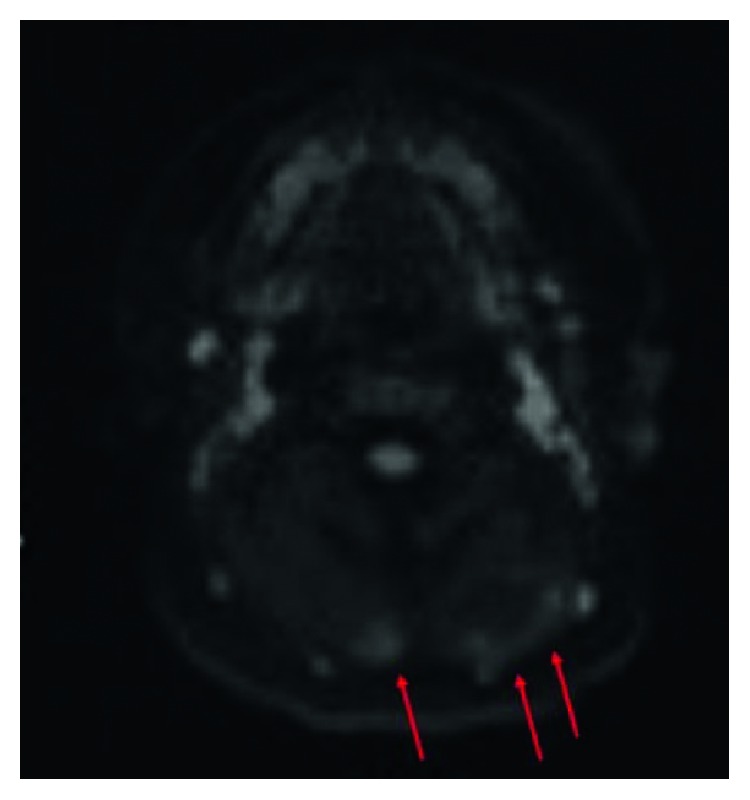
Axial diffusion-weighted imaging (DWI) demonstrates areas of rounded restricted diffusion in the posterior left and right paraspinal muscles of the neck (arrows).

**Figure 4:**
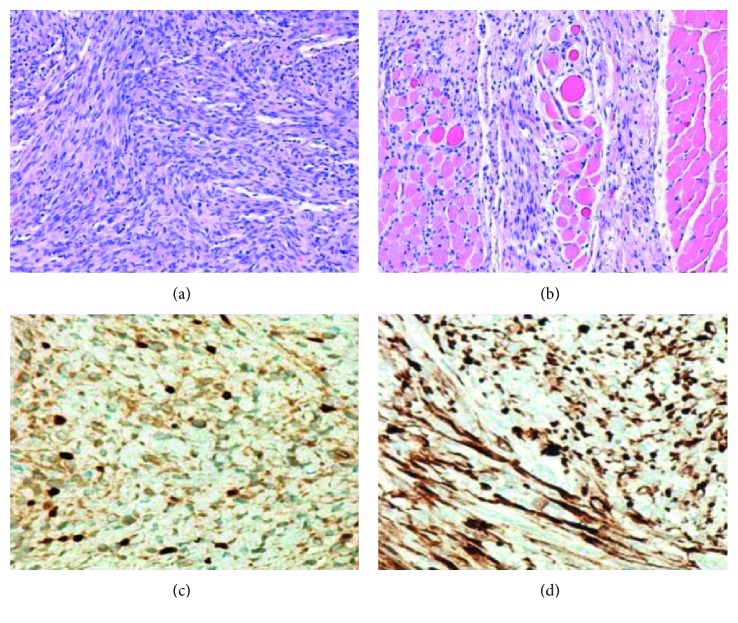
(a, b) H&E stain at medium magnification show both discrete nodules of spindle cells (a) as well as individual spindle cells infiltrating among variably atrophic skeletal muscle fibers and collagenized stroma (b). Mitoses were conspicuous, and there was no anaplasia or significant pleomorphism. (c, d) Immunohistochemistry stains demonstrate scattered nuclear positivity with myogenin (c) and strong cytoplasmic positivity with desmin in a majority of cells (d). The Ki-67 proliferative index was around 40%.

A follow-up ultrasound one month later showed stability of the mass in terms of size and imaging appearance. An MRI performed at six weeks old (Figures 2 and 3) showed an ill-defined, mildly enhancing mass with areas of increased T2 signal and diffusion restriction. The mass was located within and immediately posterior to the paraspinal musculature, predominantly on the left. The mass was biopsied and then surgically excised. Microscopy revealed a spindle cell and sclerosing rhabdomyosarcoma (Figure 4). Fluorescence in situ hybridization (FISH) studies revealed the presence of a VGLL2 gene rearrangement. No abnormalities were reported in the NCOA2 gene. An adjacent lymph node was also biopsied and was negative for malignancy. A PET examination revealed no evidence of metastatic disease. The patient received chemotherapy following surgery and is doing well on two-year follow-up.

## 3. Discussion

Rhabdomyosarcoma is a malignant neoplasm of mesenchymal origin which shows variable differentiation into striated skeletal muscle. Although relatively common in children, representing up to 7% of all pediatric malignancies, the diagnosis of congenital RMS in the neonatal period is rare. This case is unique in that the mass was present in utero as well as at the time of birth.

Rhabdomyosarcoma has a variety of clinical presentations, the most common being a rapidly growing soft tissue mass. Depending on the location, the tumor may cause a range of clinical symptoms related to mass effect and obstruction. Tissue sampling is usually required to make a definitive diagnosis.

Historically, RMS had been divided into three subtypes: embryonal, alveolar, and pleomorphic. The spindle cell variant was included within the embryonal category along with botryoid and anaplastic variants. Recent evidence, however, suggests that the spindle cell and sclerosing RMS may represent a discrete morphologic spectrum from the embryonal subtype because of similar clinical presentations and histologic features [7]. Specifically, there is a subtype of spindle cell variant which displays areas of prominent hyaline sclerosis and a pseudovascular growth pattern, overlapping with the morphology of sclerosing RMS. As a result, the 2013 WHO classification merged the spindle cell and sclerosing subtypes into a single pathologic entity with a possible shared pathogenesis [5]. The spindle cell subtype, as seen in our patient, demonstrates a high degree of skeletal muscle differentiation and therefore has been shown to have low malignant potential [8].

Much work has been done on identifying gene rearrangements associated with the different subtypes of RMS. Alaggio et al. recently described a novel VGLL2 gene rearrangement which was found to be the most common genetic abnormality in congenital/infantile spindle cell RMS. VGLL2 is involved in regulating muscle-specific gene transcription and is exclusively expressed in skeletal muscle in the adult. Other genetic alterations include the NCOA2 gene fusion, which is common in congenital cases, and the MYOD1 gene mutation, which tends to occur in older children and adults. They found that cases which harbor gene fusions involving VGLL2 and NCOA2 had a favorable clinical outcome, whereas tumors associated with mutations in the MYOD1 gene showed a more aggressive course with a higher mortality despite multimodal therapy [7].

## 4. Conclusion

Here, we describe a case of congenital rhabdomyosarcoma manifesting as a neck mass at birth. Because of its location on the posterior neck, our differential diagnosis included fibromatosis colli and solitary infantile myofibroma. Histologic sampling offered a definitive diagnosis and further characterized the tumor as the spindle cell variant of RMS. Furthermore, through FISH studies, we were able to identify the VGLL2 gene rearrangement which has been shown in recent studies to harbor a favorable clinical outcome.
